# Identification and experimental validation of biomarkers associated with T cell infiltration in psoriasis

**DOI:** 10.1186/s41065-026-00707-5

**Published:** 2026-07-04

**Authors:** Li Zhang, Xiao-Yan Yang, Fa-Zeng Li, Zhi-Quan Liu, Lei Zhang, Zheng-Fa Ou

**Affiliations:** 1 Department of Dermatology, Kunming First People’s Hospital, 1228 Beijing Road, Panlong District, Kunming, Yunnan 650051 China; 2 Department of Dermatology, Yan’an Hospital Affiliated To Kunming Medical University, Kunming, Yunnan China

**Keywords:** Psoriasis, T cells, Bioinformatics, Biomarkers, Immune infiltration

## Abstract

**Background:**

Psoriasis represents a prevalent long-term inflammatory skin condition marked by the dysregulation of immune responses. T cells are integral to the pathogenesis of psoriasis. This study conducted a comprehensive analysis to recognize biomarkers associated with T cell infiltration in psoriasis and to elucidate their underlying molecular mechanisms.

**Results:**

Three biomarkers (AKR1B10, C10orf99, and CKS2) demonstrated high sensitivity and specificity in receiver operating characteristic (ROC) curve, and the reliability of the developed nomogram diagnostic model was observed. In addition, gene set enrichment analysis (GSEA) revealed notable enrichment of the biomarkers in the NOD-like receptor signaling pathway and focal adhesion. Immune infiltration analysis indicated elevated levels of activated B cells and CD8 T cells in psoriasis samples, with the biomarkers showing meaningful correlations with the majority of immune cell infiltration statuses. Importantly, preliminary reverse transcription quantitative PCR (RT-qPCR) validation showed increased expression of AKR1B10, C10orf99, and CKS2 in psoriasis tissues, confirmed higher expression of AKR1B10, C10orf99, and CKS2 in psoriasis patients.

**Conclusion:**

AKR1B10, C10orf99, and CKS2 may serve as candidate molecules for future mechanistic studies and provide potential diagnostic biomarkers for further investigation of psoriasis-related immune regulation.

**Supplementary Information:**

The online version contains supplementary material available at https://doi.org/10.1186/s41065-026-00707-5.

## Background

Psoriasis is a chronic systemic inflammatory disorder characterized by a relapsing clinical course, whose onset and progression are jointly driven by genetic susceptibility and environmental factors. Approximately 2%−3% of the world's population is affected by psoriasis [1]. The typical clinical manifestations of psoriasis are scaly erythema or plaques, which not only affect the appearance and social life of patients, but also make psoriasis patients susceptible to complications such as depression, psoriatic arthritis and cardiometabolic syndrome, which in turn affect the quality of life and life expectancy of patients [2].The risk factors associated with psoriasis are highly complex, with environmental factors such as air pollutants, infections, unhealthy lifestyles and various cardiovascular diseases all increasing the risk of psoriasis [3]. Nevertheless, abnormalities in immune system activity, genetic factors, and environmental elements may all be involved in the pathogenesis of psoriasis, and psoriasis remains a challenging disease. Accordingly, the search for biomarkers are of great importance for diagnosis and research into the molecular mechanisms of psoriasis.

T cells occupy a pivotal position in the immunopathogenesis of psoriasis. The type 1 helper T cells (Th1), type 17 helper T cells (Th17), and type 22 helper T cells (Th22) subsets of helper T (Th) cells each promote disease progression via their respective cytokine cascades, whereas dysfunction of regulatory T cells (Treg) contributes to a breakdown in immune regulation [4]. In genetically susceptible individuals, exposure to relevant triggers prompts antigen-presenting cells (APCs) to release pro-inflammatory mediators—including IL-23, IFN-α, and IL-22—which activate naive T cells and direct their differentiation toward distinct Th cell subsets, ultimately resulting in the abundant production of cytokines such as TNF, IFN-γ, IL-17, and IL-22. These subset-specific cytokines then act on keratinocytes to perpetuate the inflammatory cycle characteristic of psoriasis [5]. Furthermore, immune memory is manifested in the skin, and tissue-resident memory T cells show a rather high degree of infiltration in psoriasis, thereby inducing psoriasis lesions [6]. It is now widely accepted in the clinical community that psoriasis is a multigenic disease mediated by T cells and dendritic cells. Therefore, it is imperative to identify the biomarkers associated with T cell infiltration in psoriasis, to elucidate the mechanism of its development and progression, and to search for better and novel diagnostic modalities.

This investigation, based on the transcriptome data of psoriasis, uses methods such as weighted gene co-expression network analysis (WGCNA) and machine learning to discriminate the biomarkers associated with T cell infiltration in psoriasis. It examines the functions and interactions among the biomarkers, investigates their immune properties and molecular pathways, and provides further information for differential diagnosis, with the expectation of providing a theoretical foundation and scientific principles for clinical application and further investigation of psoriasis.

## Material and methods

### Data preparation

Psoriasis-related gene expression data were retrieved from the Gene Expression Omnibus (GEO) database (http://www.ncbi.nlm.nih.gov/geo/), covering the GSE13355 and GSE14905 datasets. In GSE13355 (platform: GPL570), 58 involved skin samples from psoriasis cases, labeled as “PP”, and 64 normal skin samples from healthy controls were selected as the training set. According to the original study, involved skin biopsies were obtained from psoriatic plaques, while normal skin biopsies were obtained from healthy controls. In GSE14905 (platform: GPL570), lesional skin samples from patients with psoriasis and healthy control skin samples were used for validation; the original study reported that the psoriasis samples were derived from plaque-type psoriasis.

### Differential expression analysis

Psoriasis-associated DEGs were identified by applying the "Limma" package (v 1.38.0) [7]. to compare gene expression profiles between psoriasis and normal groups within GSE13355, with screening criteria set at |log_2_FC|> 2 and *p* < 0.05. The resulting DEGs were visualized through a volcano plot and a heatmap, constructed with the "ggplot2" package (v 3.3.6) [8] and the "heatmap3" package [9], respectively.

### WGCNA

First, ssGSEA was performed using the gene set variation analysis (GSVA) algorithm to estimate T cell infiltration scores based on the previously published 28 immune cell signatures reported by Charoentong et al. [10]. These scores were then used as phenotypic traits for WGCNA. Differences in ssGSEA-derived T cell infiltration scores between the psoriasis and normal groups were compared using the Wilcoxon test and visualized with boxplots. Subsequently, these ssGSEA scores were treated as phenotypic traits to construct a WGCNA network using the “ WGCNA” package (v 1.71) [11], which was widely used in the association analysis of phenotypic traits and genes. Initially, samples were introduced into a hierarchical clustering method to remove outliers based on ssGSEA scores. Secondly, an optimal soft threshold was identified to ensure the network construction conformed to a scale-free topology. Gene adjacency was then calculated, which led to computing gene similarity. This similarity was used to derive a gene dissimilarity coefficient, ultimately resulting in the construction of a hierarchical clustering tree of genes. The minModuleSize was set to 300 and mergeCutHeight was set for 0.25 following the standards of the dynamic tree cutting algorithm. Finally, cor values between key modules and ssGSEA scores were calculated and visualized using a heatmap (|cor|> 0.3 and *p* < 0.05). To further obtain genes that were strongly associated with T cell infiltration, we performed a within-module analysis. Genes with module member (MM) > 0.2 were selected as key module genes associated with T cell infiltration scores.

### Identification, function analysis and protein–protein interaction (PPI) network of candidate genes

Candidate genes were obtained by taking the overlap between key module genes and DEGs. To elucidate the biological roles and pathway involvement of these candidates in psoriasis pathogenesis, GO and KEGG enrichment analyses were carried out using the "ClusterProfiler" package (v 4.10.1) [12], with *p* < 0.05 as the significance threshold. GO analysis enabled functional annotation across three dimensions-biological process (BP), cellular components (CC) and molecular functions (MF)-while KEGG profiling highlighted the major metabolic and signal transduction pathways in which these genes participate.

Moreover, to understand the PPI network of the candidate genes, these candidate genes were then introduced into the search tool for the retrieval of interacting genes (STRING) database (http://www.string-db.org/) to establish a PPI network, with a confidence score threshold > 0.4, to understand their interactions better.

### Machine learning screening for identifying feature genes

Least absolute shrinkage and selection operator (LASSO) regression and support vector machine-recursive feature elimination (SVM-RFE) are two widely adopted machine learning approaches in bioinformatics for dimensionality reduction and feature selection. Given the complexity of psoriasis-related gene expression data, both methods were applied to GSE13355 to systematically screen for feature genes relevant to psoriasis and T cell biology. For LASSO regression, the "glmnet" package (v 4.1–8) [13],was used; the core principle of this method lies in penalizing regression coefficients by introducing a regularization parameter λ, with cross-validation performed to evaluate model performance across different λ values. The configuration associated with the lowest binomial deviance was regarded as the optimal model, from which feature genes were extracted. For SVM-RFE, the "caret" package (v 6.0–94) [14] was adopted; this method constructs a classifier and ranks genes by their contribution weights, recursively excluding those with the lowest scores until a stable, high-discriminability gene set is obtained. The feature genes identified by both LASSO and SVM-RFE were then intersected to yield the final feature gene list.

### Validation of biomarkers by expression levels and receiver operating characteristic (ROC)

Wilcoxon test was applied to examine whether feature genes exhibited concordant expression differences between normal and psoriasis groups across both GSE13355 and GSE14905 (*p* < 0.05); genes meeting this criterion were designated as biomarkers, with their expression distributions illustrated via box line plots. The ROC curve for each biomarker was subsequently generated using the "pROC" package (v 1.18.5) [15], to quantify diagnostic performance, with the area under curve (AUC) serving as the primary metric.

### Gene set enrichment analysis (GSEA) of biomarkers

To probe the underlying biological processes through which biomarkers may participate in T cell-infiltrated inflammatory microenvironment of psoriasis, Spearman correlation coefficients between each key gene and the remaining genes in the training set were computed, and candidate pathways for each biomarker were ranked in descending order of correlation magnitude. GSEA was subsequently performed on these ranked gene lists via the "clusterProfiler" package (v 4.10.1) (*p* < 0.05), using "c2.cp.kegg.v7.4.symbols.gmt" from MSigDB as the reference gene set; pathways satisfying |normalized enrichment score (NES)|> 1 were considered significantly enriched, and the five most prominent results per biomarker were retained for display. Inter-biomarker correlations were additionally assessed through the "psych" package [16] and rendered graphically using the "corrplot" package (v 0.92) [17].

### Immune infiltration analysis

T cells were often closely linked to the activity of the immune apparatus. Characterizing the immune cell landscape offers a meaningful perspective for understanding psoriasis pathogenesis. Accordingly, the proportions of 28 immune cell subsets [18] in GSE13355 were quantified via the ssGSEA algorithm. Wilcoxon test was then applied to identify immune cell types whose abundance differed significantly between psoriasis and normal groups (*p* < 0.05), and only those reaching statistical significance were carried forward. Spearman's correlation analysis was further conducted to examine the inter-relationships among the differentially abundant immune cells, as well as the associations between each biomarker and these immune cell subsets; correlation pairs with |r|> 0.3 and *p* < 0.05 were regarded as biologically meaningful.

To explore the cell-type-specific expression patterns of biomarkers in psoriatic skin, the public single-cell RNA-seq dataset GSE162183 was analyzed using the “Seurat” package. Cells were filtered using the following criteria: nCount_RNA < 50,000, 200 < nFeature_RNA < 7,000, and percent.mt < 20%. After normalization and identification of highly variable genes, PCA, clustering, and UMAP visualization were performed. Principal components were selected based on JackStraw and ElbowPlot analyses. Cell types were manually annotated according to canonical marker genes from previous studies. The expression patterns of biomarkers across annotated cell types were visualized using DotPlot, FeaturePlot, and violin plots, and group differences within each cell type were evaluated using the Wilcoxon test.

### Chromosomal, subcellular localization analysis, and drug prediction

The location information of the biomarkers was downloaded from the ENSEMBL database (https://asia.ensembl.org/index.html). Subcellular localization prediction of the protein products encoded by the key genes was performed using the Genecards database (https://www.genecards.org/). Furthermore, the DSigDB database (https://dsigdb.tanlab.org/) was employed to screen for drugs associated with the biomarkers. To identify high-confidence candidates, we further selected the top five drugs with a adj.p < 0.05 and the highest Combined.Score, and then generated a gene-drug association Sankey diagram.

### Experimental validation by reverse transcription quantitative PCR (RT-qPCR)

Preliminary validation of biomarker expression was performed using RT-qPCR. Skin tissue specimens were collected from 5 patients with plaque psoriasis and 5 control subjects at the First People's Hospital of Kunming. Detailed sampling information and clinical characteristics, including sex, age, sampling site, disease duration, BSA, PASI score, and treatment information, are provided in Table S1. All participants were given informed consent. This study was conducted in accordance with the provisions of the Declaration of Helsinki. Ethical approval has been obtained from Kunming First People's Hospital Medical Ethics Committee, with the approval number: YLSH (Dan) −2025–022-01. Total RNA was isolated using Trizol reagent (Ambion, Texas, USA). RNA was reverse-transcribed into cDNA using the SweScript First Strand cDNA synthesis kit (Servicebio, Wuhan, China) following the manufacturer's recommended protocol. Quantitative PCR was carried out in a 10 μL reaction volume with 2 × Universal Blue SYBR Green qPCR Master Mix (Servicebio, Wuhan, China) on a CFX Connect Real-time Quantitative Fluorescence PCR Instrument (Bio-Rad, USA). Biomarker mRNA levels were quantified using the 2^−ΔΔCt^ method, with GAPDH serving as the internal reference gene for normalization. All primer sequences used for reverse transcription quantitative polymerase chain reaction (RT-qPCR) were synthesized by Sangon (Shanghai, China) and are listed in Table 1.

**Table 1:** The primer sequences

Primer	Sequence
AKR1B10-F	GAGTGGAAGCCTGATGTCCC
AKR1B10-R	AGGCCCTCCAGTTTCTGTTG
C10orf99-F	TCCAGCCTGCTCTGTATCCT
C10orf99-R	GCTTTGGGAGTGCTACACCT
CKS2-F	TCTTCGCGCTCTCGTTTCAT
CKS2-R	TGGACACCAAGTCTCCTCCA
GAPDH-F	ATGGGCAGCCGTTAGGAAAG
GAPDH-R	AGGAAAAGCATCACCCGGAG

### Statistical analysis

Bioinformatics data processing was performed in R language software (v 4.2.2), and graphical outputs from cell experiments were generated with GraphPad Prism (v 5.0). The relative mRNA expression levels of biomarkers were compared between groups using the t-test, while non-parametric comparisons in bioinformatics analyses were assessed by the Wilcoxon test; a p-value below 0.05 was taken as the threshold for statistical significance in all analyses.

## Results

### Recognition of 95 differentially expressed genes (DEGs) and 1,744 key module genes

**Fig. 1:**
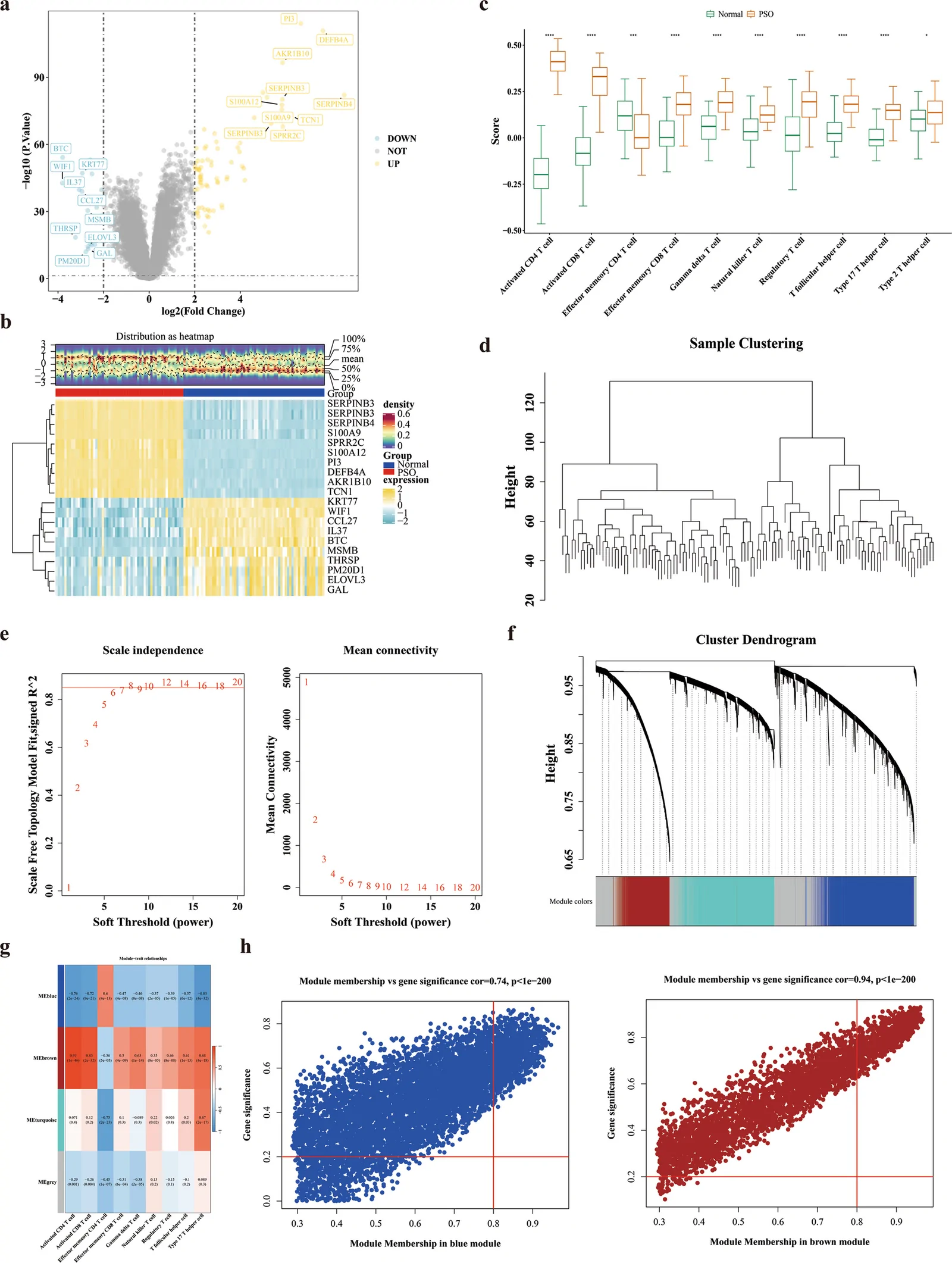
Recognition of 95 DEGs and 1,744 key module genes. **A** Volcano plot of DEGs. **B** Heat map of DEGs. **C** T cell infiltration ssGSEA score between the psoriasis and normal groups. **D** Cluster diagram of WGCNA analysis. **E** Soft threshold was determined to be 8. **F** Dendrogram of gene modules. **G** Correlation heatmap between modules and T cell infiltration ssGSEA scores. **H** Scatter diagram of key module genes related to T cells

By means of differential expression analysis in GSE13355, a total of 95 DEGs were identified, including 75 up-regulated genes and 20 down-regulated genes in the psoriasis group compared with the control group. The top 10 most markedly regulated genes in both directions were highlighted in the volcano plot and heatmap (Fig. 1A, B). Subsequently, a notable difference in T cell infiltration ssGSEA score emerged between the psoriasis and normal groups (*p* < 0.05) (Fig. 1C), indicating a potential association between T cell infiltration and psoriasis pathogenesis. No notable outliers were detected among the samples through cluster analysis, confirming their suitability for downstream analyses (Fig. 1D). A soft-thresholding power of β = 8 was selected for WGCNA because the scale-free topology fit index reached R^2^ > 0.85 while the mean connectivity remained acceptable (Fig. 1E). A co-expression network was then constructed, clustering genes into 4 distinct modules (including the grey module) (Fig. 1F). Finally, the correlation heatmap between modules and T cell infiltration ssGSEA scores (Fig. 1G) revealed notable correlations with the MEblue and MEbrown (|cor|> 0.30, *p* < 0.05). Through MM analysis, 1,744 genes were identified as key module genes with significant associations to T cell functional scores, with the MEbrown module showing the strongest correlation (cor = 0.94, *p* < 1e-200) and the MEblue module also reaching high significance (cor = 0.74, *p* < 1e-200) (Fig. 1H).

### Functional analysis of candidate genes

**Fig. 2:**
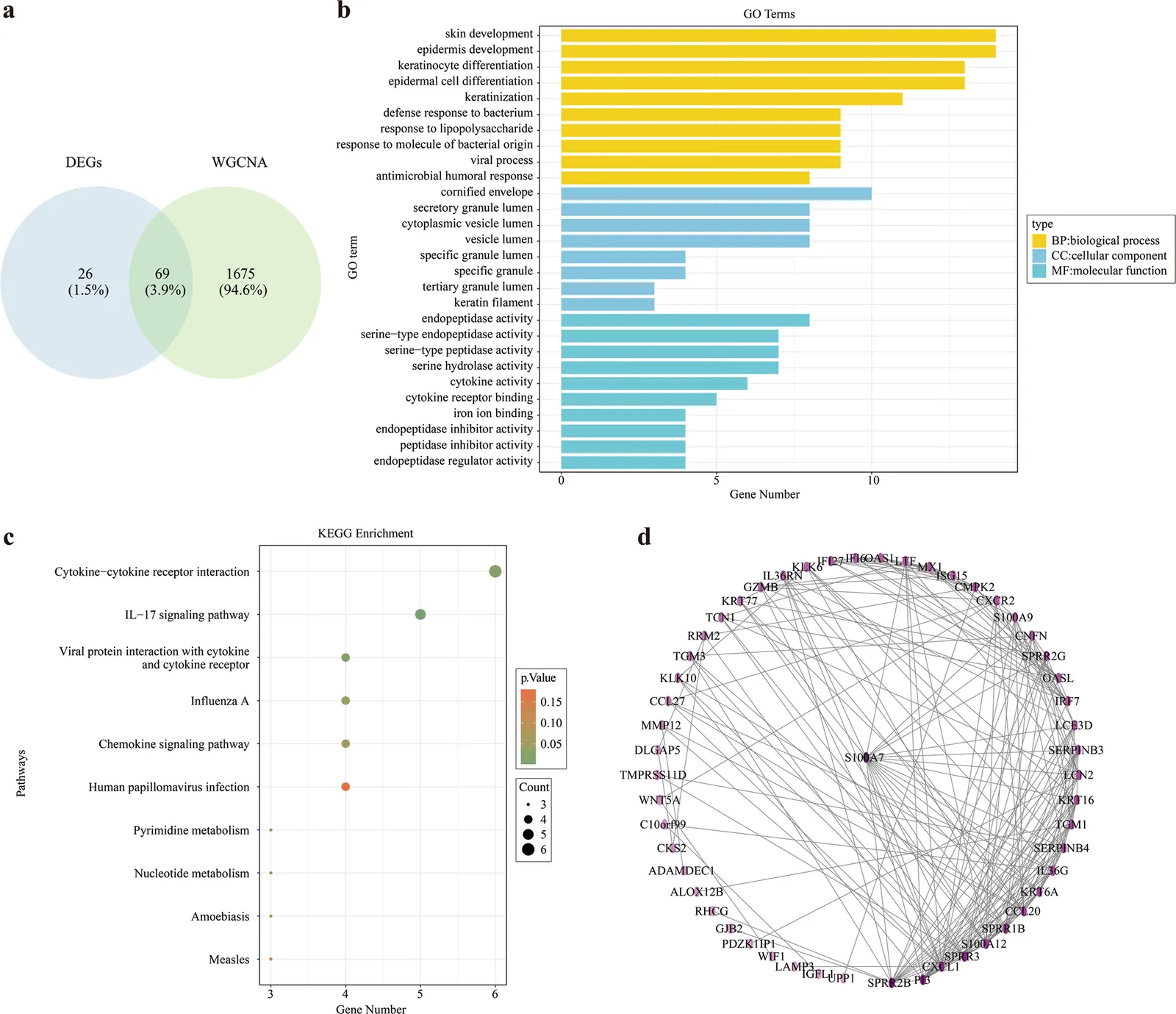
Functional analysis of candidate genes. **A** The Venn diagram illustrated The overlap between 95 DEGs and 1,744 key module genes. **B** GO analysis showed 145 GO biological functions were enriched. **C** KEGG analysis showed 17 pathways were enriched in the candidate genes. **D** The protein interaction network among the candidate genes

Venn diagram analysis was used to visualize the intersection of 95 DEGs and 1,744 key module genes, yielding 69 candidate genes for further investigation (Fig. 2A; Table S2). To characterize the molecular biological activities distinguishing the psoriasis group from the normal group, GO and KEGG enrichment analyses were performed on these 69 candidates. Across 145 enriched GO terms, skin development and epidermis development emerged as prominent entries among the 119 GO-BPs; cornified envelope and cytoplasmic vesicle lumen were represented within the 8 GO-CCs; and endopeptidase activity along with serine-type endopeptidase activity ranked among the most enriched terms across 18 GO-MFs (Fig. 2B). At the pathway level, 17 KEGG pathways were enriched, with the top 10 including IL-17 signaling pathway, influenza A, and cytokine − cytokine receptor interaction (Fig. 2C**)**. Protein–protein interaction (PPI) network construction (confidence > 0.4) further revealed that S100A7 maintained extensive connectivity with other candidate genes (Fig. 2D), offering additional perspective on the molecular features associated with psoriasis and providing candidate genes for further mechanistic investigation.

### The identification and validation of biomarkers

**Fig. 3:**
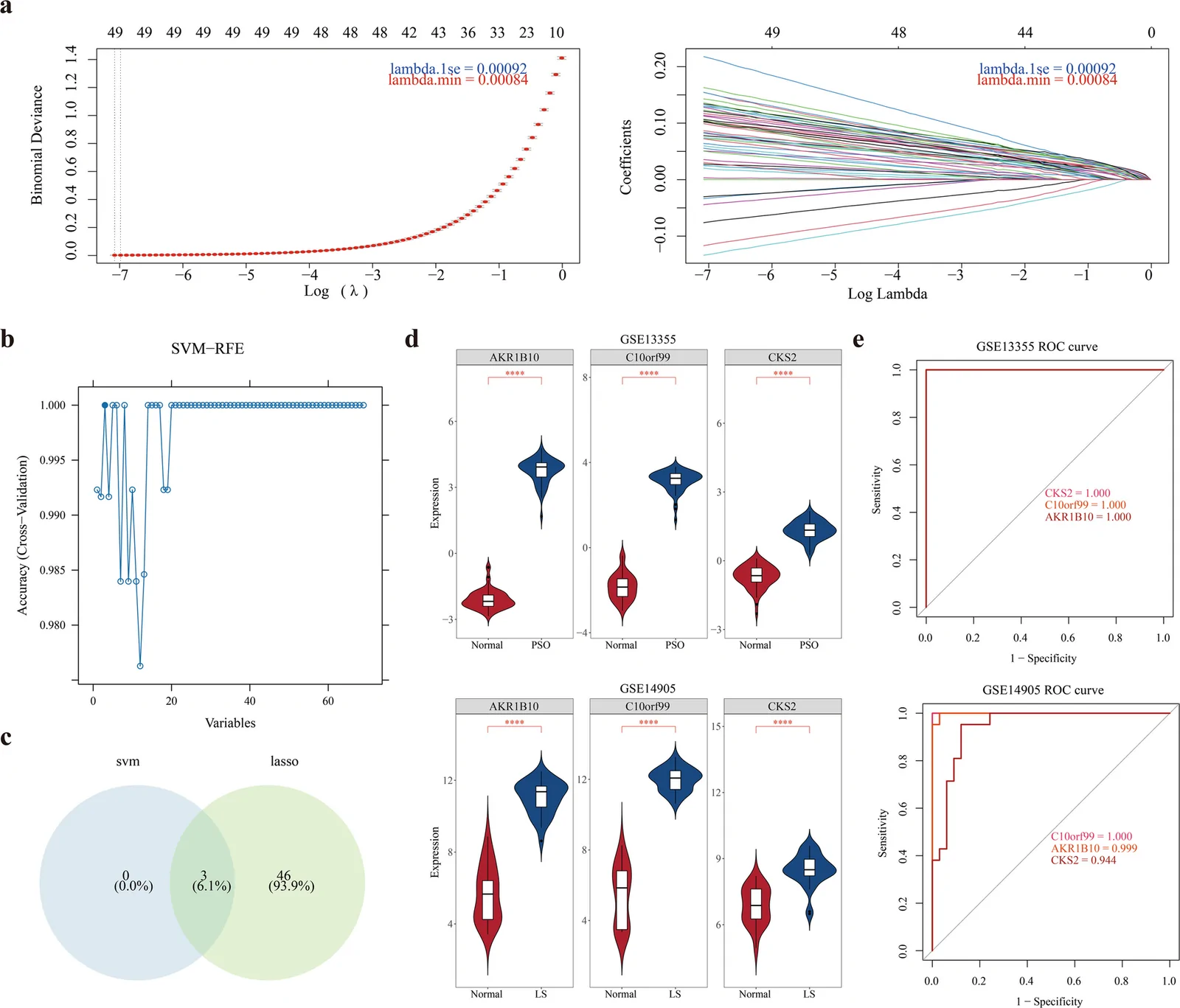
The identification and validation of biomarkers. **A** LASSO regression analysis, including the cross-validation curve for selecting the optimal λ and the coefficient profile plot. **B** SVM-RFE feature selection curve showing cross-validation accuracy across different numbers of variables. **C** The LASSO analysis and SVM-RFE analysis were screened to cross the feature genes of psoriasis related to T cells, resulting in 3 biomarkers, namely AKR1B10, C10orf99, and CKS2. **D** AKR1B10, C10orf99, and CKS2 were up-regulated in the psoriasis group in GSE13355 and GSE14905. **E** ROC validation showed all AUCs of biomarkers were greater than 0.9 in GSE13355 and GSE14905

LASSO and SVM-RFE algorithms were employed in combination to screen for feature genes relevant to both psoriasis and T cells. In LASSO regression, the cross-validation curve was used to determine the optimal regularization parameter, and 49 feature genes were retained at lambda.min = 0.00084 and lambda.1se = 0.00092 (Fig. 3A; Table S3). SVM-RFE was then performed to further reduce feature redundancy. The cross-validation accuracy curve showed that the model achieved optimal performance when three variables were retained, and these genes were AKR1B10, C10orf99, and CKS2 (Fig. 3B). Taking the intersection of feature genes identified by both algorithms yielded 3 psoriasis-related biomarkers connected to T cells: AKR1B10, C10orf99, and CKS2 (Fig. 3C). Expression validation across GSE13355 and GSE14905 confirmed that all three were consistently up-regulated in the psoriasis group relative to normal controls, with statistically significant differences (*p* < 0.05) (Fig. 3D). ROC curve analysis further substantiated their diagnostic value, as AUC values for AKR1B10, C10orf99, and CKS2 all exceeded 0.9 in both datasets (Fig. 3E), reinforcing the reliability of these three genes as psoriasis biomarkers.

### GSEA of AKR1B10, C10orf99, and CKS2

**Fig. 4:**
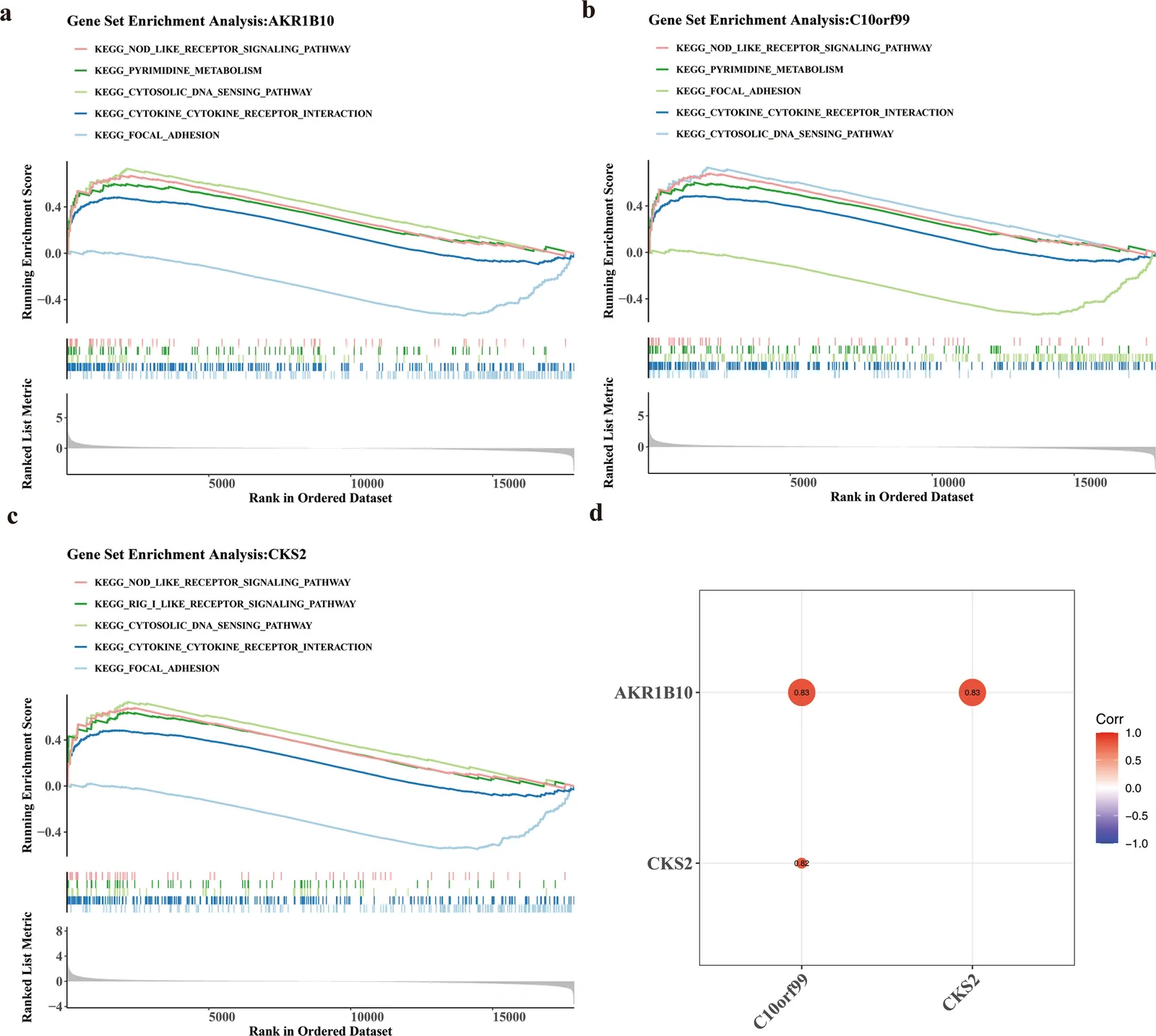
GSEA of AKR1B10, C10orf99, and CKS2. **A** The pathways significantly associated with AKR1B10 in the analysis of GSE13355. **B** The pathways significantly associated with C10orf99 in the analysis of GSE13355. **C** The pathways significantly associated with CKS2 in the analysis of GSE13355. **D** The correlation analysis demonstrated a strong correlation among the three biomarkers (cor > 0.8, *p* < 0.05)

GSEA enables systematic identification of coordinated transcriptional changes across biological pathways via gene set enrichment strategies. In GSE13355, AKR1B10 showed significant enrichment in the NOD-like receptor signaling pathway, pyrimidine metabolism, and focal adhesion (Fig. 4A). C10orf99 was predominantly enriched in the NOD-like receptor signaling pathway, pyrimidine metabolism, and focal adhesion (Fig. 4B). CKS2-associated gene sets were significantly enriched in the NOD-like receptor signaling pathway and focal adhesion (Fig. 4C). Additionally, the correlation analysis demonstrated a strong correlation among the three biomarkers (correlation coefficient (cor) > 0.8, *p* < 0.05) (Fig. 4D). These results deepen the understanding of the biological functions mediated by these biomarkers in psoriasis.

### Immune infiltration in psoriasis

**Fig. 5:**
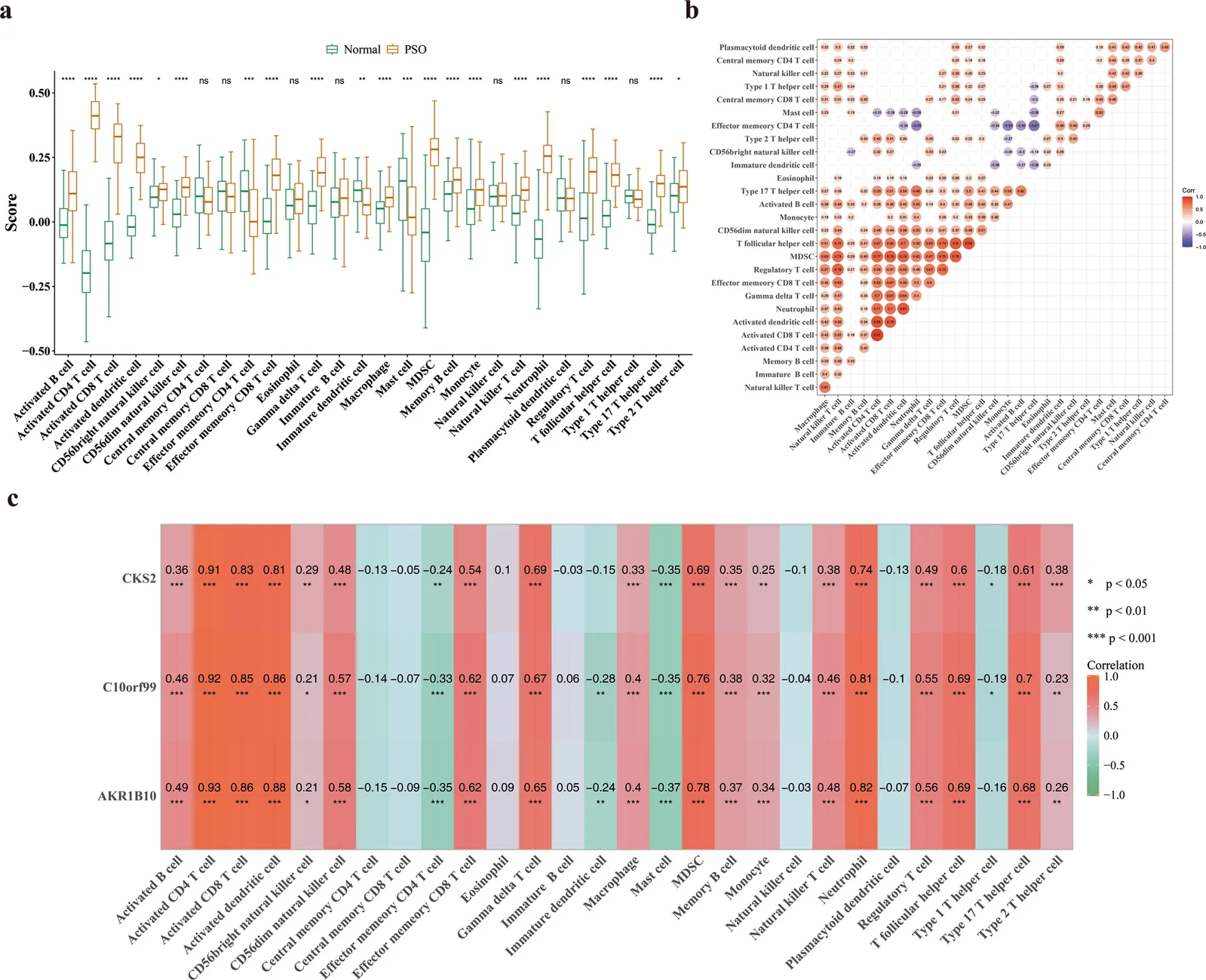
Immune infiltration in psoriasis. **A** Enrichment scores revealed a considerable difference in 21 types of immune cells between the psoriasis and control group. **B** The correlations between differential immune cells. **C** The association between the biomarkers and the differential immune cells

To characterize the immune microenvironment in psoriasis, immune cell infiltration was further analyzed. Immune infiltration analysis identified significant differences in 21 immune cell subtypes between the psoriasis and control groups, including activated B cells, activated CD4 T cells, and CD56dim natural killer cells (*p* < 0.05) (Fig. 5A). Inter-cellular correlation analysis showed that activated CD4 T cells were positively correlated with activated CD8 T cells (cor = 0.91, *p* < 0.05) and activated dendritic cells (cor = 0.84, *p* < 0.05), whereas type 17 T helper cells exhibited a marked negative correlation with effector memory CD4 T cells (cor = − 0.61, *p* < 0.05) (Fig. 5B). Additionally, strong associations were detected between the three biomarkers and differential immune cells; activated CD4 T cells, activated dendritic cells, activated CD8 T cells, and the three biomarkers all demonstrated notably high positive correlations (cor > 0.80, *p* < 0.05) (Fig. 5C). These results highlight the differential immune infiltration landscape and its linkage with key biomarkers, offering new perspectives for elucidating psoriasis pathogenesis.

To further clarify the cellular context of AKR1B10, C10orf99, and CKS2 in psoriatic skin, single-cell RNA-seq analysis was performed using GSE162183. After quality control, 18,295 cells and 18,642 genes were retained for downstream analysis (Figure S1A). Unsupervised clustering identified 24 cell clusters, which were annotated into eight major cell types based on canonical marker genes, including keratinocytes, T cells, dendritic cells, fibroblasts, endothelial cells, smooth muscle cells, mast cells, and epithelial cells (Figure S1B-D). Cell-type-specific expression analysis showed that AKR1B10, C10orf99, and CKS2 were not restricted to T cells. Instead, these genes showed relatively higher expression in keratinocytes, while differential expression between psoriasis and control samples was also observed in several cell types, including keratinocytes, endothelial cells, T cells, and dendritic cells (*P* < 0.05) (Figure S1E-G). These results suggest that the three candidate biomarkers may reflect transcriptional changes in the immune-inflammatory microenvironment of psoriatic skin rather than T cell-specific expression.

### Localization information and targeted drugs of biomarkers

**Fig. 6:**
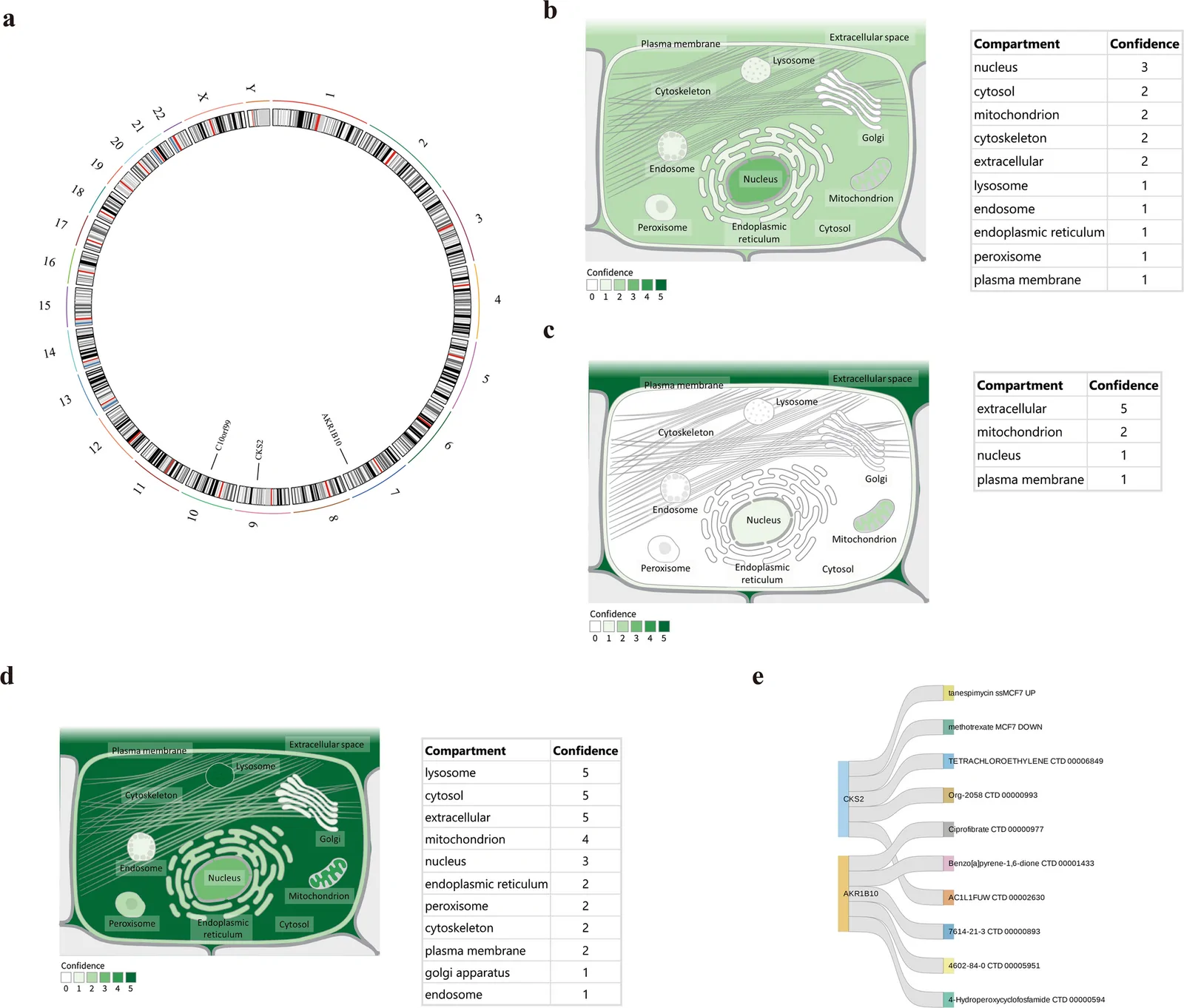
Localization information and targeted drugs of biomarkers. **A** Chromosomal localization map. **B**-**D** Subcellular localization maps of CKS2 (**B**), C10orf99 (**C**), and AKR1B10 (**D**). On the left side of the images are schematic diagrams of cellular localization results; on the right side are the confidence scores for each subcellular region. The color scale in the bottom-left corner indicates that the darker the color, the higher the confidence of the localization in that region. **E** Network diagram of the association between drugs and biomarkers

Chromosomal localization results revealed that CKS2 is mapped to chromosome 9, C10orf99 to chromosome 10, and AKR1B10 to chromosome 7 (Fig. 6A). Subcellular localization refers to the specific intracellular distribution of a certain protein or its expression product. The results indicated that the C10orf99 gene is predominantly localized in the extracellular space, the CKS2 gene is mainly in the nucleus, and the AKR1B10 gene is primarily distributed in lysosomes, the cytoplasmic matrix, and the extracellular space (Fig. 6B-D). To identify potential drugs targeting these key genes for disease treatment, the DSigDB database was used for drug prediction: 66 drugs were predicted for CKS2, 4 for C10orf99, and 83 for AKR1B10. The top-ranked drugs by Combined.Score include tanespimycin, methotrexate, ciprofibrate, benzo [16] pyrene-1,6-dione, AC1L1FUW, and 4-hydroperoxycyclophosphamide (Fig. 6E).

### Experimental validation of AKR1B10, C10orf99 and CKS2

**Fig. 7:**
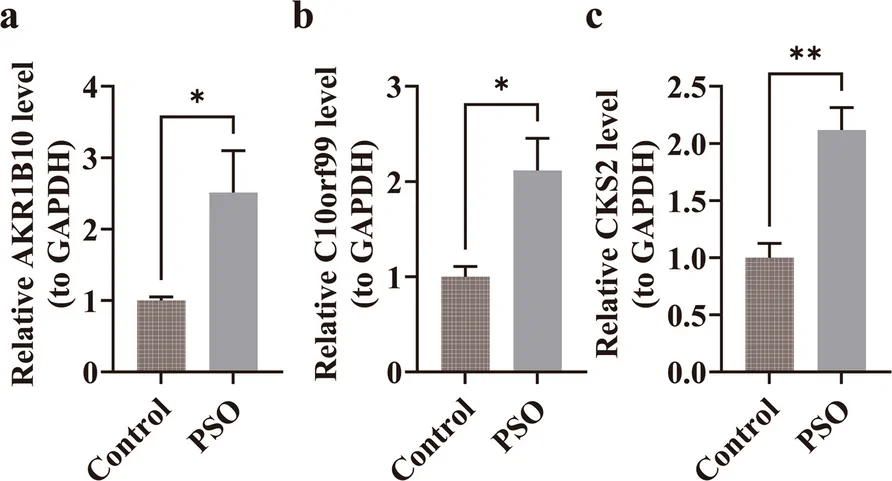
RT-qPCR results of AKR1B10, C10orf99 and CKS2 between psoriasis patients and control groups. **A** The mRNA expression level of AKR1B10 was elevated in psoriasis patients. **B** The mRNA expression level of C10orf99 was elevated in psoriasis patients. **C** The CKS2 expression level of C10orf99 was elevated in psoriasis patients

In order to preliminarily validate the expression difference of biomarkers AKR1B10, C10orf99 and CKS2 in psoriasis patients, RT-qPCR results exhibited that compared to the control group, mRNA expression levels of AKR1B10, C10orf99 and CKS2 were remarkably elevated in tissue samples from psoriasis patients (*p* < 0.05) (Fig. 7A-C). Melting peak analysis was further performed to evaluate the specificity of RT-qPCR amplification. The melt peak curves showed dominant amplification peaks for AKR1B10, C10orf99, CKS2, and GAPDH, supporting the specificity of the RT-qPCR products (Figure S2). These results preliminarily supported the upregulated expression of AKR1B10, C10orf99, and CKS2 in psoriasis tissues, suggesting their potential value as diagnostic biomarkers.

## Discussion

Psoriasis constitutes a prevalently manifested chronic inflammatory skin disorder, the pathogenic mechanism whereof is convoluted and encompasses deviations in multiple cell types and immune regulatory pathways [19]. Amongst these, T cells assume an exceedingly crucial role in the pathogenic course of psoriasis [20]. They propel the persistence of psoriatic skin inflammation and disease advancement by discharging a substantial quantity of inflammatory factors, interacting with other immune cells, and engaging in the immune regulatory network of the skin tissue microenvironment [10, 21]. In this study, we identified three candidate markers AKR1B10, C10orf99 and CKS2 associated with T cell infiltration levels via WGCNA, machine learning screening, external validation, immune infiltration analysis and preliminary RT-qPCR verification. Although AKR1B10 and C10orf99 have been previously reported to be involved in psoriasis-related inflammation and keratinocyte proliferation [22–24], the clinical implications of the three-gene panel consisting of AKR1B10, C10orf99 and CKS2 in diagnostic modeling and its correlation with immune infiltration remain poorly elucidated. Accordingly, the main value of this study lies in the identification and preliminary validation of a marker panel linked to T cell infiltration, which provides a reference for subsequent mechanistic research and the development of diagnostic biomarkers for psoriasis.In the present study, with the help of machine learning algorithms and expression validation, three T cell-related potential biomarkers (AKR1B10, C10orf99, and CKS2) associated with the pathogenesis of psoriasis were identified and found to be associated with the regulation of immune system activities based on the correlation between immune infiltration and the biomarkers, which provide new clues for the pathogenesis of psoriasis and potential diagnostic methods.

Previous studies have demonstrated that C10orf99/GPR15L is highly expressed in psoriatic lesions and may promote keratinocyte proliferation and inflammatory responses [25]. Meanwhile, C10orf99/GPR15L is also associated with immune cell recruitment and T cell-mediated immune regulation [26]. CKS2 is primarily involved in the regulation of cell cycle and cell proliferation [27], which may contribute to the pathological feature of excessive keratinocyte proliferation in psoriatic lesions. AKR1B10 has been reported to be linked to inflammatory diseases and the production of pro-inflammatory factors mediated by NF-κB [24, 26, 28]. Collectively, these three genes may reflect the pathological processes of psoriasis through distinct yet interrelated pathways, including keratinocyte activation, proliferation remodeling and immune inflammation regulation.The main significance of this study lies not in elaborating the known functions of each individual gene, but in identifying a three-gene signature with potential diagnostic and immunological value. AKR1B10, C10orf99 and CKS2 were consistently upregulated in both the training and validation datasets and were collectively incorporated into the nomogram model, which exhibited favorable diagnostic efficacy. In addition, the three markers were positively correlated with multiple immune cell subsets, particularly activated CD4⁺ T cells, activated CD8⁺ T cells and activated dendritic cells. These findings suggest that the combined expression pattern of AKR1B10, C10orf99 and CKS2 can more comprehensively characterize the immune and inflammatory profiles of psoriatic lesions compared with a single biomarker.

Single-cell analysis further clarified the cellular distribution of these three biomarkers. AKR1B10, C10orf99 and CKS2 were not specifically expressed in T cells. On the contrary, they exhibited relatively high expression levels in keratinocytes, and differential expression was also detected in several immune and stromal cell types. This finding is consistent with the pathological features of psoriatic lesions, where crosstalk between activated keratinocytes and infiltrating immune cells sustains local inflammation. Therefore, these genes should be regarded as candidate biomarkers associated with immune infiltration that reflect the inflammatory skin microenvironment, rather than T cell-derived or T cell-specific genes.

GSEA further reveals the regulatory roles of these genes in multiple pathways, thereby illuminating their influence on psoriasis. NOD-like receptor (NLR), as an intracellular protein and a member of pattern recognition receptors, is capable of triggering multiple signaling pathways and plays a core role in both innate and adaptive immunity [24]. NLR forms signaling complexes, which are capable of giving rise to the activation of transcription factors or effector caspases, and through the activation of inflammasomes, result in the occurrence of inflammatory arthritis [28]. Accumulating evidence from prior research indicates that ferroptosis and necroptosis are critically involved in the onset and progression of psoriasis. The genes related to ferroptosis and necroptosis are significantly enriched in immune-related and inflammatory pathways and might exert their effects through the NLR signaling pathway [26, 29]. This study also disclosed that AKR1B10, C10orf99, and CKS2 are conspicuously enriched in the NLR signaling pathway in psoriasis.Accumulating evidence has indicated that inflammasome components such as NLRP1 and NLRP3 are involved in the inflammatory responses of psoriasis. They can amplify the inflammatory cascade between keratinocytes and immune cells by facilitating the release of inflammatory factors including IL-1β and IL-18 [30]. From a biological perspective, AKR1B10 has been reported to be associated with inflammatory disorders and the production of pro-inflammatory factors driven by NF-κB. Notably, NF-κB signaling acts as an initiating signal for NLR-related inflammatory responses [27, 31]. C10orf99/GPR15L modulates inflammatory responses in keratinocytes and skin barrier formation, and may participate in the remodeling of the local inflammatory microenvironment [32]. CKS2 primarily regulates cell cycle and cell proliferation. Its upregulation may reflect the aberrant proliferation and cellular stress of keratinocytes in psoriatic lesions [23].Collectively, these three genes do not directly regulate NLR proteins. Instead, they indirectly participate in the NLR-mediated inflammatory amplification network via inflammatory activation, keratinocyte responses and cell proliferation. Further functional experiments are required to clarify their exact regulatory mechanisms in future studies.

As intracellular pattern recognition receptors, RIG-I-like receptors (RLR) are responsible for sensing viral or bacterial nucleic acids and triggering innate immune responses in the host. Notably, the evasion of immune responses mediated by RLRs could potentially lead to persistent infections, host immune deficiencies, and carcinogenesis [33]. The DDX58 gene, which encodes the Retinoic acid inducible-gene I (RIG-I) protein, has been established as a susceptibility gene for psoriasis. Once activated, RIG-I generates IL-23, giving rise to the occurrence of mouse psoriasis-like skin diseases. Research findings have indicated that the dysregulation of the antiviral immune response of the host via innate pattern recognition receptors could potentially constitute the pathophysiological basis of psoriasis [34].

Immune infiltrated cells unveil the considerable roles that multiple immune cells exert in psoriasis. B cells are activated upon antigen stimulation and with the assistance of T cells and other cells; activated B cells can produce antibodies, participate in the humoral immune response, and play a role in immune defense. In addition, activated B cells can produce various cytokines, including TNF-α and IL-6 [35]. TNF-α and IL-6 can facilitate the activation of T cells, the proliferation of keratinocytes, and the infiltration of inflammatory cells, thereby aggravating the condition of psoriasis. In the immune pathogenesis of psoriasis, there exists a complex interaction between T cells and B cells [36]. Activated T cells are capable of furnishing signals to facilitate the activation and differentiation of B cells. Meanwhile, the antibodies and cytokines generated by activated B cells can further regulate the functions of T cells [37]. This interaction forms a positive feedback loop, exacerbating the inflammatory response in psoriasis.

In addition, the elevated ssGSEA score of CD56dim natural killer (NK) cells suggests that the activation of innate immunity is implicated in the pathogenesis of psoriasis. Previous studies have demonstrated that NK cells and related innate lymphoid cell subsets contribute to psoriasis pathogenesis by secreting inflammatory cytokines and interacting with keratinocytes and other immune cells [38]. Notably, recent flow cytometry results have revealed that CD56dimCD16⁺ NK cells are correlated with the PASI scores of psoriasis patients [36]. Accordingly, the alterations in activated B cells and CD56dim NK cells observed in our study may reflect a broader immune activation beyond T cell responses. Nevertheless, these results are inferred from bulk transcriptomic data and require further validation using multiple approaches, including immunohistochemistry, immunofluorescence, flow cytometry and single-cell sequencing.

In addition, activated CD4 + T cells can differentiate into various effector subsets-Th1, Th2, Th17, and Treg cells-each of which possesses specific functional characteristics and cytokine secretion patterns [20, 39]. Among these, Th1 and Th17 subsets are particularly implicated in psoriasis pathogenesis, as their differentiation drives the abundant production of pro-inflammatory cytokines such as IFN-γ, TNF-α, and IL-17. These cytokines are capable of facilitating the proliferation and activation of keratinocytes and endothelial cells, and recruiting other inflammatory cells to the site of the skin lesion, thereby amplifying the inflammatory response.

In the research, three biomarkers (C10orf99,CKS2,and AKR1B10) were identified, which were associated with the pathogenesis of psoriasis. Experimental validation further confirmed the elevated expression of the three biomarkers in psoriasis tissue samples. Subsequent functional enrichment analysis revealed prominent enrichment of these biomarkers in the NLR signaling pathway, focal adhesion, and RLR signaling pathway, all of which have been demonstrated to be critically involved in psoriasis pathogenesis. Immune infiltration similarly disclosed significant dissimilarities in the presence of 21 immune cells engaged in the inflammatory response between psoriasis tissues and the control group, particularly a positive correlation between activated CD4 T cells, CD8 T cells, dendritic cells, and the three biomarkers.

This study has several limitations. First, this study was mainly based on public microarray datasets and RT-qPCR validation with a small sample size, which may lead to platform bias, insufficient sample size and overestimation of diagnostic performance. Given the substantial transcriptomic differences between psoriatic lesions and normal skin, the diagnostic value of the identified markers still needs to be verified in larger, prospective cohorts with higher clinical heterogeneity.Secondly, the demographic data and information regarding psoriasis subtypes in public GEO datasets are incomplete. Although all subjects in the RT-qPCR validation cohort were diagnosed with plaque psoriasis, further validation is required to determine whether these findings are applicable to other subtypes such as pustular psoriasis and guttate psoriasis.Third, AKR1B10, C10orf99 and CKS2 should be regarded as candidate biomarkers associated with T cell infiltration and the inflammatory skin microenvironment, rather than T cell-specific genes. Although single-cell analysis was performed in this study, their spatial localization and protein expression still need to be verified by spatial transcriptomics, immunohistochemistry or immunofluorescence assays.In addition, the results of immune infiltration were inferred via ssGSEA and have not been directly validated by immunohistochemistry, immunofluorescence or flow cytometry.Lastly, neither functional assays nor drug experiments were conducted in this study. Accordingly, these genes can only be considered candidate molecules for subsequent mechanistic research rather than validated therapeutic targets. Future studies shall further explore their functional mechanisms through gene knockdown, gene overexpression, Western blot analysis and animal models.

## Conclusion

In conclusion, this study identified AKR1B10, C10orf99 and CKS2 as diagnostic biomarkers correlated with T cell infiltration in psoriasis. Their upregulation was validated in two GEO datasets and preliminary RT-qPCR experiments. Further functional investigations are required to elucidate their biological roles in the pathogenesis of psoriasis.

## Supplementary Information

Supplementary Material 1: Figure S1. Single-cell RNA-seq analysis of AKR1B10, C10orf99, and CKS2 in psoriatic skin. (A) Quality control of GSE162183 single-cell RNA-seq data. (B) UMAP visualization of unsupervised cell clusters. (C) UMAP visualization of annotated cell types. (D) Dot plot of canonical marker genes used for cell-type annotation. (E) Expression levels of AKR1B10, C10orf99, and CKS2 between psoriasis and control samples across different cell types. (F) Dot plot and feature plots showing the expression distribution of AKR1B10, C10orf99, and CKS2 across annotated cell types. (G) Feature plots showing the expression of AKR1B10, C10orf99, and CKS2

Supplementary Material 2: Figure S2. Melting curve analysis of RT-qPCR products. (A) AKR1B10. (B) C10orf99. (C) CKS2. (D) GAPDH

Supplementary Material 3: Table S1. Clinical characteristics of psoriasis patients and control subjects used for RT-qPCR validation

Supplementary Material 4: Table S2. 69 candidate genes acquired for subsequent analysis

Supplementary Material 5: Table S3. 49 LASSO-feature genes

## Data Availability

The public datasets used for bioinformatics analysis in this study are available from the GEO database (http://www.ncbi.nlm.nih.gov/geo/) under accession numbers GSE13355 and GSE14905. Additionally, skin tissue specimens were collected from 5 psoriasis patients and 5 control subjects at the First People's Hospital of Kunming. The RT-qPCR validation data generated in this study are available from the corresponding author upon reasonable request.The raw data and analysis code generated in this study have been deposited in the Figshare database under the accession number:https://doi.org/10.6084/m9.figshare.31315489.

## Abbreviations

Th: Helper T
Th1: Type 1 helper T cells
Th17: Type 17 helper T cells
Th22: Type 22 helper T cells
Treg: Regulatory T
APCs: Antigen-presenting cells
GEO: Gene Expression Omnibus
GSVA: Gene set variation analysis
PPI: Protein-protein interaction
GSEA: Gene set enrichment analysis
GO: Gene Ontology
BP: Dimensions-biological process
CC: Cellular components
MF: Molecular functions
STRING: Search tool for the retrieval of interacting genes
LASSO: Least absolute shrinkage and selection operator
SVM-RFE: Support vector machine-recursive feature elimination
ROC: Receiver operating characteristic
AUC: Area under curve
DCA: Decision curve analysis
NES: Normalized enrichment score
RT-qPCR: Reverse transcription quantitative PCR
DEGs: Differentially expressed genes
CKS2: Cyclin-dependent kinase-regulating subunit 2
CDKs: Cyclin-dependent kinases
AKR1B10: Aldo ketoreductase family 1 member B10
NLR: NOD-like receptor
RLR: RIG-I-like receptors
RIG-I: Retinoic acid inducible-gene I
NK: Natural killer
